# The role of multi-walled carbon nanotubes in enhancing the hydrolysis and thermal stability of PLA

**DOI:** 10.1038/s41598-024-58755-8

**Published:** 2024-04-10

**Authors:** Judith Yareli Diaz Varela, Lucero Guadalupe Burciaga Jurado, Imelda Olivas Armendáriz, Carlos Alberto Martínez Pérez, Christian Chapa González

**Affiliations:** 1 Ingenieria Biomédica, Instituto de Ingeniería y Tecnología, Universidad Autónoma de Ciudad Juárez, 32310 Ciudad Juárez, Chihuahua Mexico; 2 Grupo de Nanomedicina, Universidad Autónoma de Ciudad Juárez, 32310 Ciudad Juárez, Chihuahua Mexico; 3 Departamento de Física y Matemáticas, Instituto de Ingeniería y Tecnología, Universidad Autónoma de Ciudad Juárez, 32310 Ciudad Juárez, Chihuahua Mexico

**Keywords:** Poly lactic acid, Carbon nanotubes, Degradation, Physiological, Thermal, Biodegradable, Biomaterials, Materials chemistry

## Abstract

Polylactic acid (PLA) is a bioresorbable and biodegradable polymer extensively used in various biomedical and engineering applications. In this study, we investigated the mass loss and thermal properties of PLA-multi-walled carbon nanotube (MWCNT) composites under simulated physiological conditions. The composites were prepared by melting PLA with 0.1, 0.5, 1.0, and 5.0 wt% MWCNTs using an ultrasonic agitator, and FTIR analysis confirmed composite formation. Subsequently, the composites were subjected to hydrolysis under simulated physiological conditions (pH 7.4 and 37 °C) for up to 60 days. The results revealed that the mass loss of the composites decreased with increasing MWCNT content, suggesting that the presence of MWCNTs decelerated the hydrolysis process. On day 58, the mass loss of pure PLA was 12.5%, decreasing to 8.34% with 0.1% MWCNT, 5.94% with 0.5% MWCNT, 4.59% with 1% MWCNT, and 3.54% with 5.0% MWCNT. This study offers valuable insights into the behavior of PLA-MWCNT composites under physiologically simulated conditions, facilitating the development of new polymer composites with enhanced thermal stability and degradation resistance for biomedical applications.

## Introduction

In the field of materials science, polymers are the building blocks of several areas due to their versatility and ease of processing. When reinforced with various materials such as metal oxides^1–3^, fibers^4–6^ or carbon nanotubes (CNTs)^7,8^, polymers gain in mechanical strength, thermal stability, and electrical conductivity, extending their utility in high-performance applications. Understanding the properties of materials and the cooperative relationship between polymers and reinforcements is decisive for designing advanced materials tailored to specific technological demands. Bioresorbable and biodegradable polymers are extensively studied for a diverse array of biomedical and pharmaceutical applications. They find utility in various sectors^9^, including drug delivery^10–12^, the fabrication of resorbable prostheses^13^, sutures^14–17^, implants^18^, and scaffolds^19–22^. This broad application scope is attributed to their remarkable versatility in terms of degradable property.

When a polymer encounters an aqueous medium, it undergoes a phenomenon known as hydrolysis or hydrolytic degradation^23^. This process entails the breakdown of the extended polymer chains due to the influence of water molecules, resulting in a decrease in molecular weight and, ultimately, causing a loss of mass in the solid polymers. Degradation initiates when the rate of water diffusion into the biopolymer is slower than the rate at which the biopolymer transforms into water-soluble materials. The degradation process unfolds in two stages: the initial stage targets the amorphous regions of the polymer matrix, which are the first to undergo degradation, followed by the second stage that involves the crystalline areas of the biopolymer. In aqueous biological environments, enzymes, primarily hydrolases, also play a role as catalysts in the degradation process of the biopolymer. Research has established that the degradation rate of polymers is directly contingent on factors such as molecular weight, crystallinity rate, the microstructure of the material, and the presence of reinforcements such as carbon nanotubes (CNTs)^24–26^.

PLA is a biodegradable and bioresorbable aliphatic polyester, which can be obtained from natural products such as corn and sugarcane^27,28^. PLA is obtained through the polymerization of lactic acid^28,29^. It has gained significant attention in recent years due to its biodegradability, which reduces its environmental impact^30–32^. Likewise, PLA and derivatives are currently widely studied for its use in medical and biological applications, 3D printing^33^, food packaging^34^, disposable products^35^, drug delivery systems^36,37^, the creation of resorbable prostheses, biodegradable sutures, implantable medical devices^26,38^, tissue engineering scaffolds^37^, and detection of biomolecules^39^. In most of these applications, the hydrolysis of PLA is an important factor to consider, as it determines the rate at which the material will degrade and be replaced by the host tissue.

What has enabled the advancement of absorbable devices is that the degradation byproducts of PLA are non-toxic^40,41^, and when naturally broken down within the body, mechanical removal is unnecessary. Given the established safety of PLA within the human body, there arises a critical necessity to enhance their mechanical robustness, especially within the context of targeted biomedical applications. A prime example is the ongoing evolution of biodegradable stents, where heightened mechanical resilience stands as an imperative requirement^42^. However, the intrinsic mechanical constraints, typified by low tensile strength^43^ and stiffness present obstacles to their optimal utilization in high-performance scenarios. To meet this challenge, scientists should strategically consider reinforcing polymers, perhaps with materials like MWCNTs^44–46^, as a broader strategy to modulate hydrolysis rates and customize the composite properties according to specific application needs. MWCNTs, composed of multiple layers of graphene sheets rolled into cylindrical shapes, exhibit exceptional mechanical, thermal, and electrical properties. Their inherent versatility renders them highly appealing across various applications, encompassing electronics^47^, energy storage^48^, and biomedicine^49–51^. Harnessing the remarkable strength, stiffness, and thermal conductivity of MWCNTs holds the potential to significantly enhance the properties of polymer matrices^52^. While the biocompatibility of multi-walled carbon nanotubes (MWCNTs) remains a subject of ongoing debate, emerging evidence suggests that their judicious utilization in specific biomedical applications could yield substantial benefits. Recent studies indicate that deliberately incorporating MWCNTs can enhance polymer properties without compromising cellular viability underscoring their promising potential in the development of advanced materials^53–59^. The effectiveness of MWCNTs in modifying mechanical, thermal and rheological properties has been demonstrated in many types of materials such as cement-based materials^60^, and polypropylene^61,62^.

Recent studies have explored the mechanical, thermal, and degradation behavior of polylactic acid (PLA) and multi-walled carbon nanotubes (MWCNTs) composites. These investigations highlight the improvement of the physical properties of PLA with the increase in CNT content, in concentrations ranging from 0.1% to 5%^63–67^. Mechanical tests indicate improved elongation at break with CNT incorporation, although tensile strength may decrease^65^. In the same vein, the addition of CNT increases the surface roughness^68^^.^. These findings underscore the assessment of PLA reinforced with MWCNTs (PLA-MWCNT) degradation holds significant importance in the realm of biomedical applications^64,69^. In this way, our study pioneers in evaluating the mass loss of PLA-MWCNT composites under simulated physiological conditions (pH 7.4 and 37 °C), offering valuable insights into their suitability for biomedical applications. In contrast to previous studies, which focus mainly on mechanical attributes or degradation conditions that do not resemble physiological ones, our research focuses on three key issues: 1) the variations in mass of PLA-MWCNT in simulated physiological solution under physiologically relevant conditions, 2) elucidating changes in morphology attributed to hydrolytic degradation over a month of study, and 3) exploring thermodynamic parameters such as T_max_ and activation energy before and after hydrolytic degradation. This distinctive focus on mass dynamics, morphological alterations, and thermodynamic shifts sets our study apart, providing comprehensive information for advancing the development of PLA/MWCNT composites in the biomedical field.

## Results and discussion

**Figure 1:**
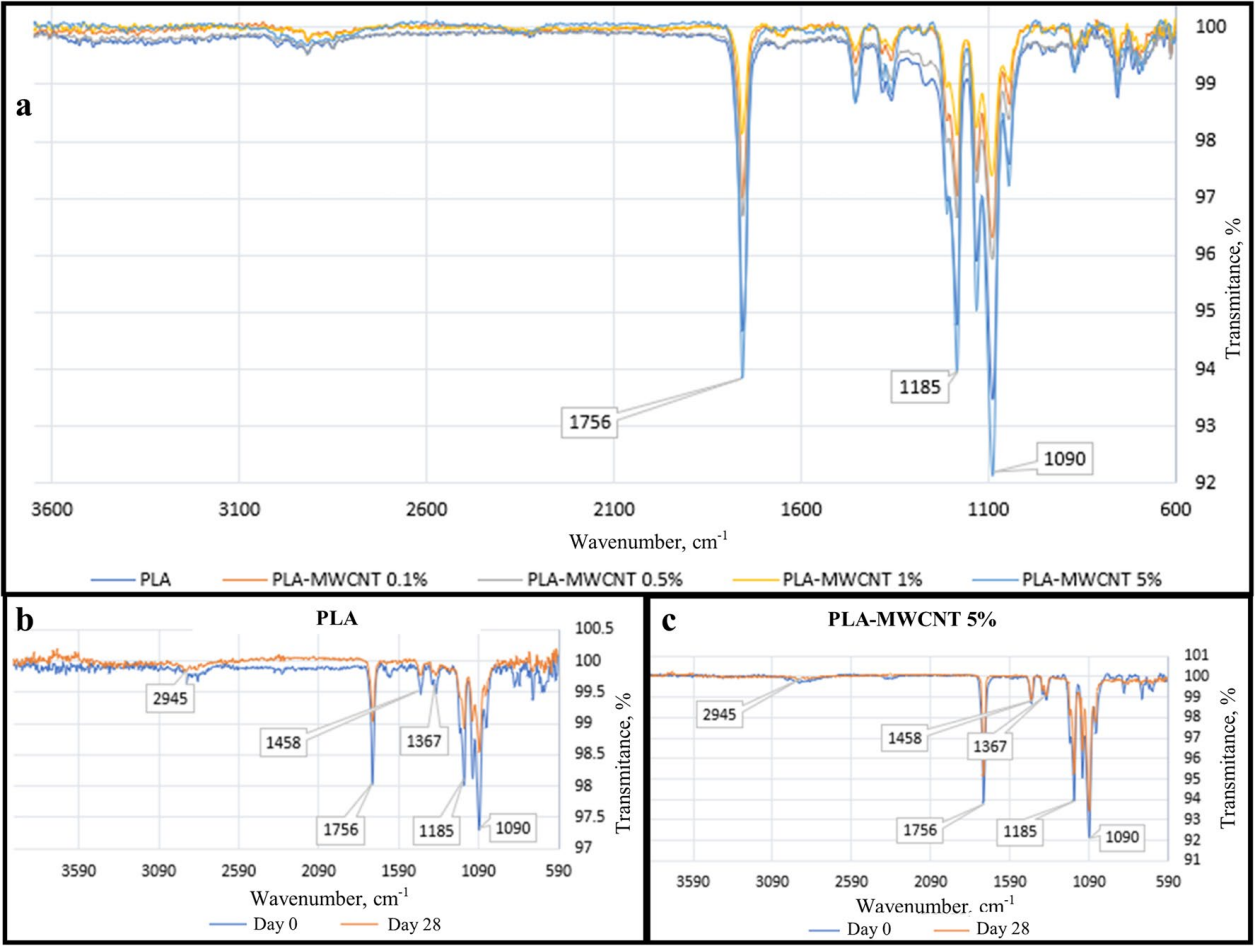
FTIR spectra of PLA-MWCNT at different mass ratios. (**a**) The spectra of PLA-MWCNT at different mass ratios, (**b**) comparison of FTIR spectra of PLA at time zero and after 28 days of treatment at 37 °C and pH 7.4, (**c**) comparison of FTIR spectra of PLA-MWCNT 5% at time zero and after 28 days of treatment at 37 °C and pH 7.4.

In Fig. 1a, the FTIR spectra depicts the variations in PLA-MWCNT formulations with mass ratios ranging from 0 to 5.0% MWCNT. Each spectrum represents the distinctive chemical fingerprint of the composite materials, showcasing changes in functional groups and molecular interactions as the MWCNT content increases. Meanwhile, Fig. 1b presents the FTIR spectrum of pristine PLA before and after 28 days of exposure to simulated physiological conditions. The observed alterations in peak intensity and position elucidate the evolving molecular structure of PLA due to degradation processes, providing valuable insights into the material's stability and behavior under physiological environments. It can be seen how the transmittance increases in some bands such as 1756 cm^−1^, 1185 cm^−1^ and 1090 cm^−1^ which are characteristic absorption band of the ester group of the carbonyl group (–C=O) at 1756 cm^−1^, the asymmetric -C-O stretching absorption band at 1090 cm^−1^ and 1185 cm^−1^. While the absorption band at 2945 cm^−1^ and 1367–1458 cm^−1^ are attributed to the stretching and bending vibrations of the -CH groups of PLA which is a sign of PLA degradation. Similarly, Fig. 1c shows the infrared spectrum of the PLA-MWCNT 5% material subjected to these same experimental conditions and highlights the variations in the aforementioned bands corresponding to the carbonyl functional group. However, it can be appreciated that the variation in transmittance also occurs, but to a lesser extent compared to PLA alone. This is attributable to the presence of the carbon nanotubes that intertwine the PLA chains and prevent water from acting on the polymeric chains.

**Figure 2:**
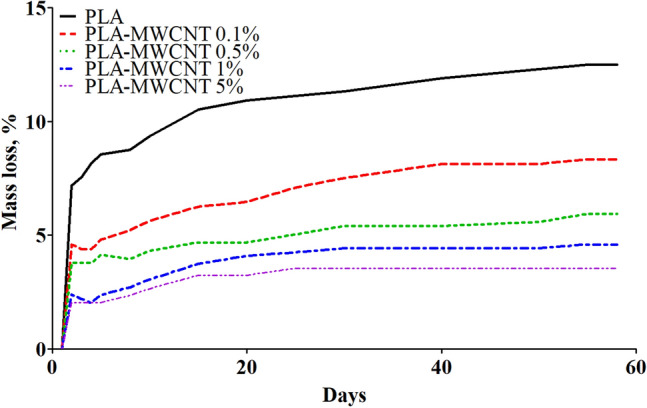
Graph showing the percentage of mass loss of PLA-MWCNT samples over time of incubation at 37 °C in PBS solution with pH 7.4

As shown in Fig. 2, the degradation rate of the composites, represented as a percentage, initially exhibited a higher rate during the first 13 days of the test, but then slowed down in the later stages. Compared to pure PLA, the degradation rate of PLA-MWCNT composites was lower throughout the entire hydrolysis process. Hydrolysis, a degradation process in which polymer chains are broken down by water molecules, resulting in the formation of small molecules such as lactic acid, is one of the main mechanisms of degradation for PLA. Factors that can influence the rate of hydrolysis include the crystallinity of the material, as crystalline domains are less permeable to water. The incorporation of MWCNTs appears to promote PLA crystallization, as evidenced by the presence of sharper and more intense diffraction peaks in the XRD patterns of the composites compared to pure PLA according to literature^65^. MWCNTs can act as nucleation sites for crystallization, increasing the degree of crystallinity of the composite and further slowing down the hydrolysis rate. Additionally, MWCNTs possess a high aspect ratio and large specific surface area, which can provide a barrier effect that slows down water penetration into the polymer matrix, thus reducing the hydrolysis rate. According to existing literature incorporating MWCNTs into PLA, ranging from 0.1 to 5%, often leads to improvements in various mechanical properties, such as tensile strength, modulus, and elongation at break^66,70–72^. The mechanical properties of the composite materials are expected to evolve during degradation. As degradation progresses, the polymer chains may undergo hydrolysis, leading to changes in the molecular structure and overall mechanical behavior of the composite. Specifically, the degradation process may alter the dispersion and interfacial bonding between MWCNTs and the polymer matrix, affecting the composite's mechanical properties.

The incorporation of MWCNTs significantly impacts the degradation behavior of PLA composites. The degradation rate, represented as a percentage mass loss, is consistently lower for PLA-MWCNT composites compared to pure PLA throughout the 58-day testing period. Notably, the pure PLA pellet exhibits a mass loss of 12.5% on day 58, while the addition of increasing MWCNT content progressively reduces mass loss, with the 5.0 wt% MWCNT composite showing a minimal loss of 3.54%. Our observations align with prior studies demonstrating a slower degradation rate for PLA reinforced with MWCNTs^73^. Comparing the observed degradation rates of PLA pristine and its nanocomposites with MWCNTs across various studies provides valuable insights into their degradation behavior under different conditions (Table 1). For instance, studies demonstrate that neat PLA degrades faster than PLA nanocomposites^63–65^, emphasizing the role of MWCNTs in enhancing the material's resistance to degradation. In the same sense, studies have shown that the incorporation of MWCNTs can increase the contact angle of PLA, indicating decreased wettability^74,75^ . The molecular orientation of materials with higher degrees of crystallinity reduces the ability of water to diffuse into the polymer matrix. For this reason, the overall effect appears to be a slower diffusion of water into the polymer matrix due to enhanced crystallinity.

**Table 1:** Comparative degradation analysis of PLA and PLA/CNTs under various environmental condition.

Material	Degradation Conditions	Degradation Rate	Ref
PLA/MWCNT (0.1%, 0.5%, 1%, and 5%)	Phosphate buffered saline (PBS) at pH 7.4 in orbital incubator at 37 °C	PLA pristine degrades faster than nanocomposites, there is a relationship between the amount of MWCNT and the rate of degradation	This study
PLA dissolved in dichloromethane and tetrahydrofuran, mixed with MWCNT–COOHs (0.5, 1, and 2.5 wt %)	Immersion in phosphate buffer solution (pH 7.2) with lipase enzymes for hydrolytic degradation	Neat PLA presented a higher weight loss compared with its nanocomposites	^63^
PLA/CNTs (0.5%, 1% and 2%) sutures	Immersion in HANKs fluid at 37 °C, simulating human circulatory system	Total degradation of PLA was 49 weeks, while with CNTs it was extended to 73 weeks	^64^
PLA and its nanocomposite films with various amounts (0.5, 1, 3 and 5%) of CNTs	UV accelerated weathering conditions for 200 h at 95% humidity and 60 °C, following ASTM G154 protocol	Lower degradation rate compared to pure PLA	^65^

**Figure 3:**
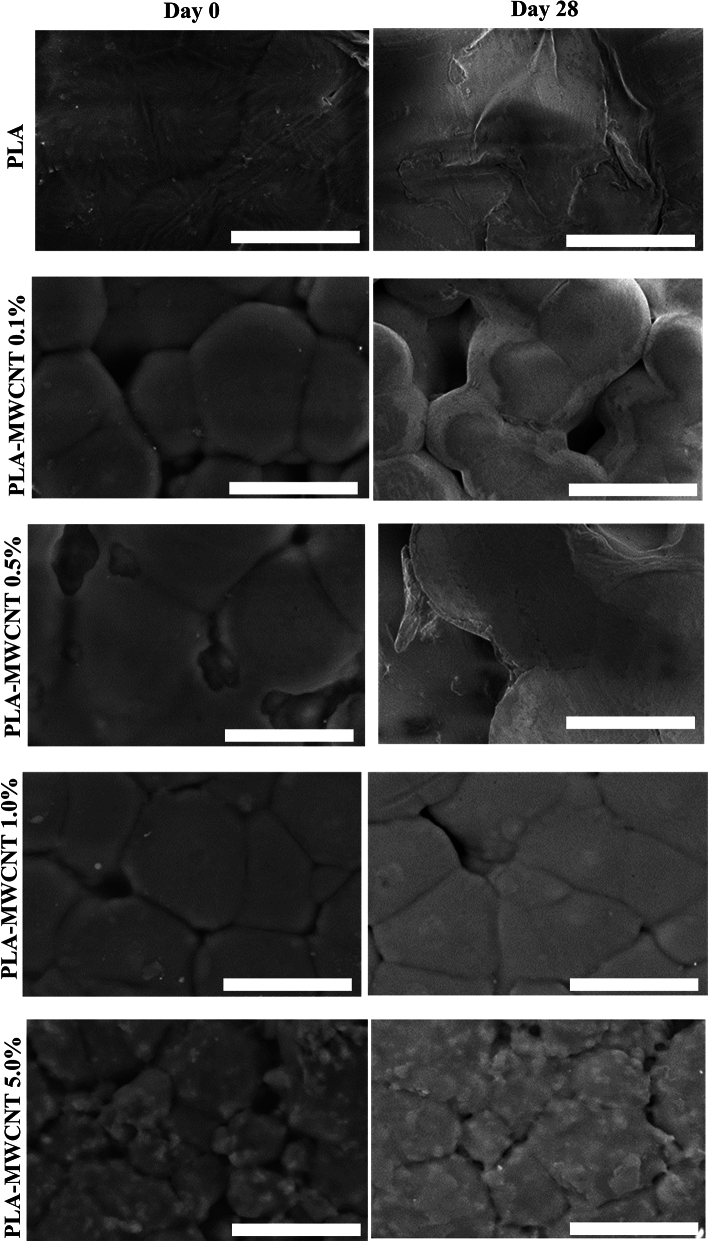
SEM micrographs of samples before and after hydrolytic degradation. The images show the surface of the initial samples (left column) compared to the samples after 28 days of incubation (right column). Scale bar 50 μm.

The morphology of the samples was analyzed using scanning electron microscopy (SEM). Figure 3 presents micrographs of the original samples and those that underwent the hydrolytic degradation process. The images indicate that the surface structure of the initial samples is distinct from that of the samples after four weeks of exposure to the experimental conditions. At lower MWCNT loadings, SEM images depict well-dispersed MWCNTs, often as individual nanotubes or small bundles. With increasing MWCNT loading, larger aggregates or clusters of MWCNTs may be observed, indicating the challenges of achieving uniform dispersion at higher concentrations. After the incubation period, deterioration and debris are evident on the PLA and PLA-MWCNT samples with lower concentrations of MWCNT (0.1%, 0.5%, and 1%). However, for samples with higher MWCNT content, which are used to enhance the polymer's strength, resulting in a delay in hydrolytic degradation, little to no degradation changes were observed after four weeks of hydrolysis. The SEM images revealed no significant qualitative changes in the appearance of the 5% PLA-MWCNT samples. However, a change in surface roughness can be observed on the PLA-MWCNT samples with 0.1%, 0.5%, and 1% MWCNT content after four weeks of hydrolysis. These results suggest that the incorporation of MWCNTs can significantly delay the degradation of PLA.

**Figure 4:**
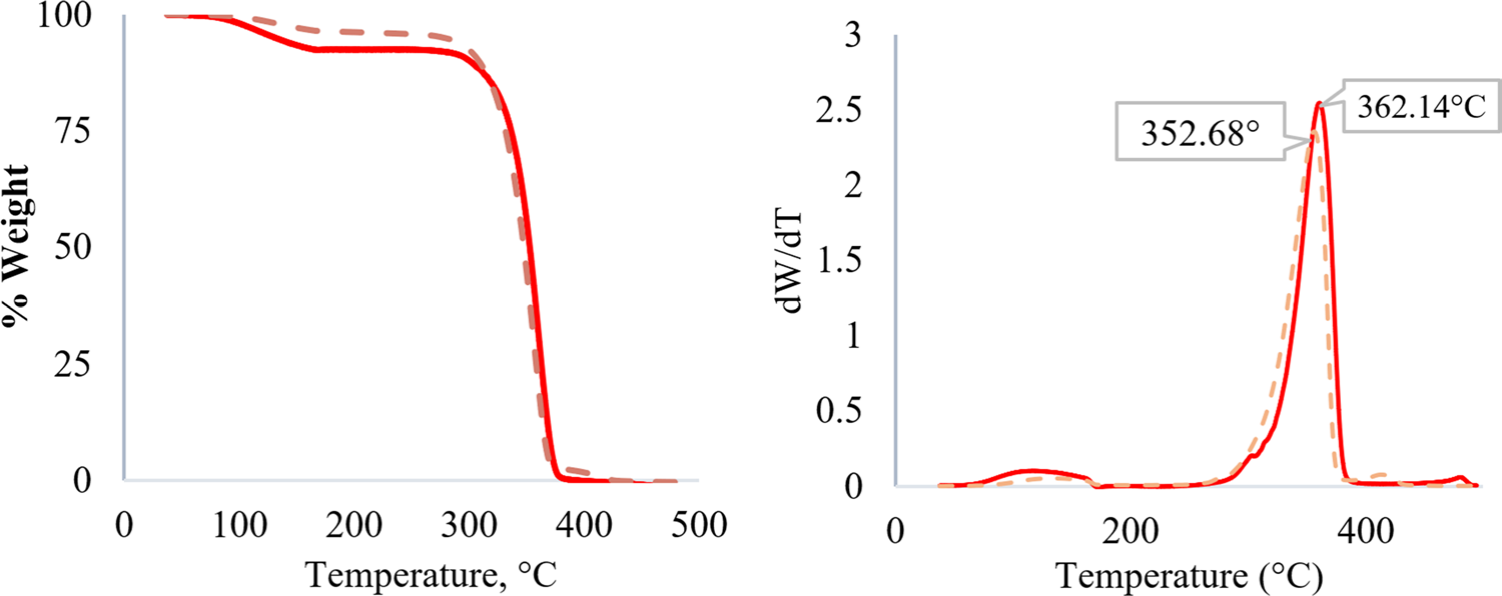
TGA of PLA-MXCNT0.1% day 0 (continuous line) and day 28 (dashed line).

**Figure 5:**
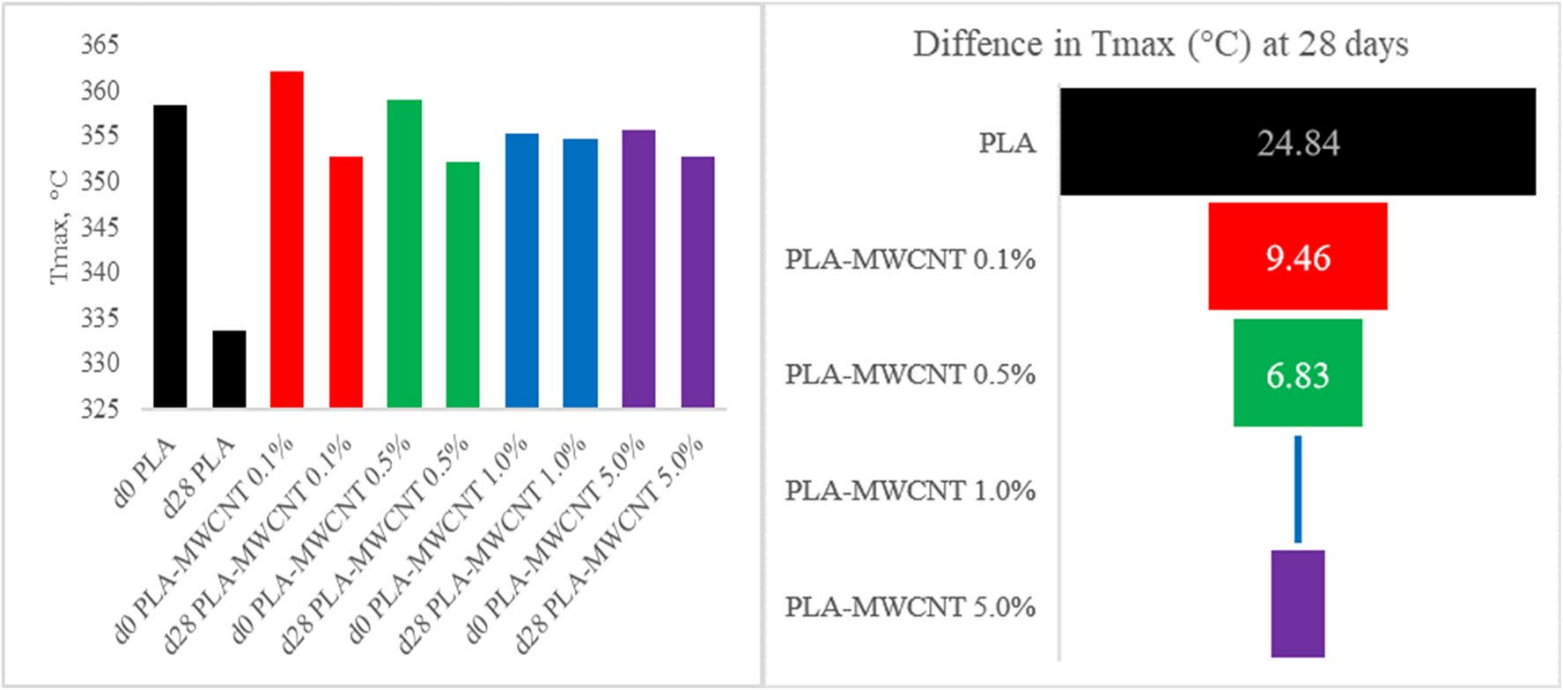
Thermal degradation behavior of the PLA-MWCNT nanocomposites with different MWCNT contents (0.1%, 0.5%, 1.0%, and 5.0%). The graph shows the decomposition temperature of the samples at time zero (fresh) and after 28 days of degradation, indicating the effect of MWCNT content on the thermal stability of the material.

To investigate the thermal stability of the nanocomposites after 4 weeks under the hydrolytic degradation process, a TGA–DSC analysis was performed. T_max_ is considered one of the key parameters governing thermal degradation behavior of PLA composites with variable composition^76^. As can be seen in Fig. 4, the TGA shows a change in the decomposition temperature for PLA-MWCNT0.1%, which went from 362.14 °C at the start of the experiment to 352.68 °C after 28 days under the experimental conditions of hydrolytic degradation. That is, the 9.46 °C difference is an indication that the PLA polymer chains have been degraded, as evidenced by the lower melting temperature of the material incubated for 28 days. This contrasts with the findings of another study^77^ which reported no significant changes in thermal properties of PLA with the addition of carbon nanotubes (CNTs). Therefore, while both studies agree on the influence of CNTs on PLA crystallinity, the contrasting observations in thermal stability could be attributed to several factors such as MWCNT type and dispersion and variations in processing techniques. Figure 5 shows the degradation temperature of all materials, where in all cases the temperature at time zero is higher than after 28 days of degradation. The difference in decomposition temperature decreases as the MWCNT content increases. However, when MWCNT5.0% is employed, the difference in decomposition temperature increases again, which can be explained by the fact that a higher MWCNT content leads to better crystallization^65^. The observed increase in thermal stability could be partially attributed to enhanced crystallinity. Compared to pure PLA, PLA-MWCNT composites exhibited superior thermal stability throughout degradation. This aligns well with other studies that suggests CNTs preserve higher molecular weight within the composite, while pure PLA shows a faster decline in decomposition temperature, indicating rapid thermal stability loss^78^.

In the case of polymer composites, the presence of fillers or additives, such as MWCNTs, can have a significant effect on the minimal energy required for material degradation, or the activation energy (Ea). The Borchardt and Daniels method enables the determination of key kinetic parameters, including Ea^79^, using data from a single DSC scan. It assumes nth order kinetics and is based on the general rate Eq. (1):

1 $$\frac{d\alpha }{dt} = k(T) {[1-\alpha ]}^{n}$$

where $d\alpha /dt$ = reaction rate (1/sec), $\alpha$ = fractional conversion, $k(T)$ = specific rate constant at temperature T, $n$= reaction order. The Borchardt and Daniels approach also assumes Arrhenius-like behavior, Eq. (2):

2 $$k\left(T\right)=Z{e}^{\frac{-Ea}{RT}}$$

where $Ea$ = Activation energy, Z = pre-exponential factor or Arrhenius frequency factor (1/s), $R$ = gas constant = 8.314 $J/mol K$. Substituting Eq. (2) in Eq. (1), rearranging and using logarithms we obtain the Eq. (3):

3 $$\frac{d\alpha }{dt} =Z{e}^{\frac{-Ea}{RT}}(1-\alpha {)}^{n}$$

4 $$ln\frac{d\alpha }{dt}={\text{ln}}\left(Z\right)-\frac{Ea}{RT}+n \,ln[1-\alpha ]$$

Equation (4) is solved through linear regression, using parameters derived from the DSC exotherm. Twenty evenly spaced temperature segments are considered, ranging from 10 to 50% of peak height and area. The Arrhenius plot of $ln[k(T)]$ versus $1/T$ confirms the validity of the second assumption, with $Ea$ and $Z$ obtained from its slope and intercept.

**Figure 6:**
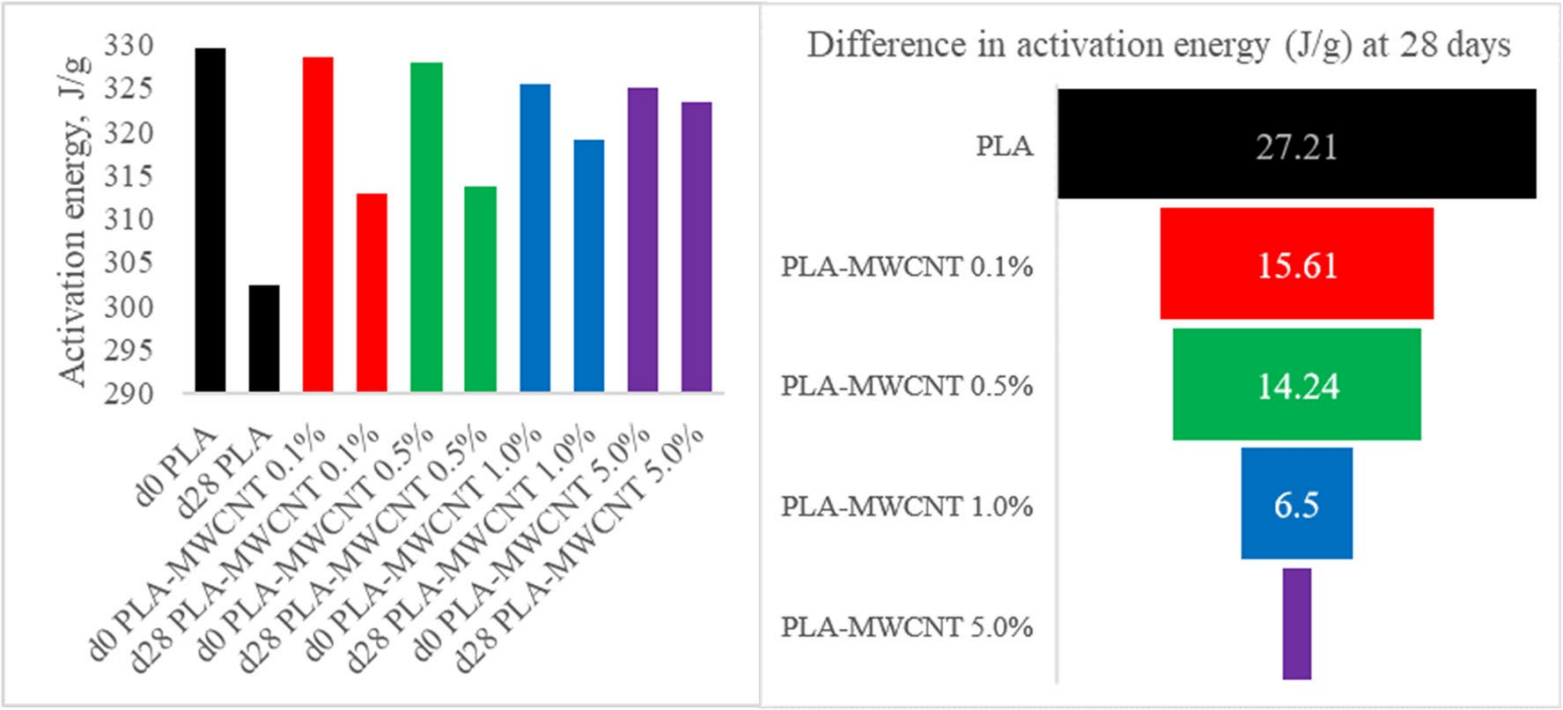
Activation energy of materials before and after hydrolysis degradation.

As shown in Fig. 6, the activation energy required for the initiation of the decomposition process decreased as the MWCNT concentration in the composite material increased. This effect can be attributed to the high thermal conductivity of CNTs, which allows the heat to disperse more easily and quickly in the polymer matrix, thus reducing the activation energy required for the decomposition process. In contrast, the activation energy was significantly reduced in pure PLA after hydrolysis, and the reduction was also observed in PLA-MWCNT, albeit to a lesser extent with increasing MWCNT content in the polymeric matrix. Moreover, it is worth noting that the hydrolytic degradation process also affects the activation energy of the polymer or the composite material. As shown in Fig. 6, the activation energy was considerably reduced in pure PLA after undergoing the hydrolysis process. In the case of PLA-MWCNT, the activation energy was also reduced, but this energy reduction was lower as the MWCNT content in the polymeric matrix increased. This suggests that the presence of MWCNTs can help to preserve the thermal stability of the material, even after undergoing the hydrolysis process.

The method outlined for fabricating PLA-MWCNT composites exhibits scalability and practical utility in real-world contexts. Its straightforward yet robust procedure, leveraging readily accessible materials and equipment, renders it amenable to large-scale implementation. This scalability augments its potential applications across diverse sectors, encompassing biomedicine and industrial realms, where the augmented characteristics of these composites confer notable benefits, such as heightened mechanical resilience and thermal endurance. Therefore, the observed slower degradation rate of PLA-MWCNT composites compared to pure PLA can be attributed to several mechanisms including enhanced crystallization, reduced water diffusion, the incorporation of MWCNTs likely creates a tortuous path for water molecules diffusing into the polymer matrix, and the strong interfacial interactions between MWCNTs and PLA chains can potentially restrict the mobility of the polymer chains, hindering their accessibility to water molecules and subsequent hydrolysis. Additionally, these interactions may offer physical protection to the adjacent PLA chains, reducing their likelihood of being hydrolyzed. While the observed changes in thermal stability after hydrolytic degradation can be explained by the cleavage of ester bonds in the PLA backbone, leading to shorter polymer chains with lower molecular weight. This decrease in molecular weight results in a reduction in thermal stability as shorter chains have weaker intermolecular forces and are more susceptible to thermal decomposition at lower temperatures.

## Conclusions

In conclusion, this study has investigated the thermal stability and degradation behavior of PLA-MWCNT nanocomposites under physiologic simulated conditions. The results of the TGA–DSC analysis showed that the MWCNT content plays a decisive role in determining the thermal stability of the material over time. This effect was attributed to the better dispersion of MWCNTs in the polymer matrix, which creates a stronger interfacial interaction, hindering the diffusion of water molecules and hydrolytic agents to the PLA chains. The activation energy required for the decomposition process was also found to decrease with the increase in MWCNT concentration, which was attributed to the high thermal conductivity of CNTs. Furthermore, our investigation revealed that at day zero, the activation energy decreases as the MWCNT content increases. However, after undergoing hydrolysis degradation for 28 days, a notable trend emerges: the activation energy shows an increasing trend with higher MWCNT concentrations. This phenomenon suggests that the presence of MWCNTs contributes to enhancing the thermal stability of the nanocomposites, particularly evident after exposure to hydrolytic conditions.

The findings of this study can provide guidance for optimizing the MWCNT content in PLA composites to achieve desired thermal stability and degradation behavior under hydrolytic conditions. The limitations of the study primarily revolved around the relatively short duration of the hydrolysis degradation study, which may not fully capture the long-term degradation behavior of the PLA-MWCNT nanocomposites under physiological conditions. Future studies can further explore changes in molar mass, thermal stability, and water sorption kinetics to elucidate the long-term degradation behavior of the composites, and mechanical properties and biocompatibility of PLA-MWCNT composites to fully understand their potential for various applications in the biomedical field.

## Materials and methods

PLA-MWCNT composites were synthesized using various concentrations of MWCNTs (0.1%, 0.5%, 1%, and 5% w/w). In selecting the concentrations of MWCNTs, we aimed to explore a broad spectrum of composite compositions to assess their impact on degradation behavior. This range encompasses concentrations commonly reported in literature^31,63–67,72^ and allows for comprehensive characterization of the material's properties. The synthesis was carried out by dissolving the PLA pellets in chloroform under constant magnetic stirring for 40–50 min at 25 °C. The beakers were covered with aluminum foil to prevent loss of solvent during the process. After the PLA were fully dissolved, the MWCNTs were added, and the mixture was allowed to rest at room temperature for 48 h to allow for complete evaporation of the chloroform. The composite pellets were then detached from the beakers by inverting them, and any residual solvent was removed by allowing the pellets to stand for an extended period of time. The initial weight of the samples was determined by allowing the samples to reach a constant weight. The PLA-MWCNT and PLA composite pellets were then placed in 24-well microplates, and 10 mg/ml phosphate buffered saline (PBS) at pH 7.4 was added, the degradation conditions were chosen based on ISO 13781:2017. The samples were kept in agitation at 37 °C, which represents human body temperature, and the mass was monitored every 24 h, changing the PBS solution every 7 days, PBS is a solution commonly used in biochemistry to imitate human extracellular fluid. The samples were dried with paper, weighed on a laboratory balance, and the average mass was calculated daily. This method described for synthesizing PLA-MWCNT composites demonstrates scalability and practical applicability in real-world scenarios. Its simple yet effective process, utilizing commonly available materials and equipment, makes it suitable for large-scale production.

FTIR was performed using a Nicolet 6700 FTIR spectrometer to analyze the chemical composition of the PLA-MWCNT composites before and after the hydrolysis tests. By comparing the FTIR spectra of the samples before and after the hydrolysis tests, it is possible to determine if there are any changes in the chemical composition of the samples as a result of the hydrolysis process, and whether the presence of MWCNTs affects the chemical composition of the composites. The number of scans and the resolution were specified as 32 and 4 cm^−1^ respectively. The samples used for the characterization were cut-out pellets of approximately 0.3cm^2^. The morphological and thermal properties of the composites were studied before and after the hydrolysis tests. The samples were analyzed using a field emission Scanning Electron Microscope (FE SEM) model JEOL JSM-7000f. The samples were dried and coated with silver for 20 s, mounted on a carbon sample holder, and analyzed using secondary electrons (SEI) and backscattered electrons (COMPO) signals. Thermogravimetric analysis (TGA) and differential scanning calorimetry (DSC) were performed using SDT Q600 equipment, with calorimetric accuracy/precision ± 2%. TGA was used to evaluate the hydrolytic degradation of PLA-MWCNT composites and compare it with neat PLA. The data obtained revealed differences in the influence of MWCNT content on degradation behavior. The samples were analyzed between 30 and 500 °C with a heating rate of 10 °C/min. The results were analyzed using TA Universal Analysis software, and the activation energy was obtained from the DSC data.

## Data Availability

The datasets generated during and/or analyzed during the current study are available from the corresponding author on reasonable request.
